# Candidiasis During Pregnancy May Result From Isogenic Commensal Strains

**DOI:** 10.1155/S1064744901000138

**Published:** 2001

**Authors:** Wayne Daniels, Douglas D. Glover, Michael Essmann, Bryan Larsen

**Affiliations:** 1 Infectious Disease Research Laboratory Des Moines University Osteopathic Medical Center 3200 Grand Avenue Des Moines IA 50312 USA; 2 Consortium on Reproductive and Developmental Health Robert C. Byrd Health Sciences Center of the West Virginia University Morgantown WV USA

## Abstract

*Objective:* Our laboratory previously demonstrated that asymptomatic vaginal colonization during pregnancy is a factor predisposing patients to subsequent symptomatic vulvovaginal candidiasis. It is unknown whether symptoms result from strain replacement or a change in host relationship to the original colonizing strain. This study was undertaken to determine whether *Candida albicans* isolates from asymptomatic women could be responsible for subsequent symptomatic vaginitis.

*Methods:* We retained isolates of *C. albicans* from women followed longitudinally through pregnancy, and identified six pairs of cultures from women who were colonized without symptoms and who later became symptomatic (average time 14 weeks). We used a random amplification of polymorphic DNA (RAPD) analysis to determine whether isolates from our study patients were genetically similar or dissimilar.

*Results:* Analysis of these pairs of yeast strains by RAPD revealed that five of the six women had symptoms apparently due to the same yeast strain that was found initially as a commensal strain. To increase the power of these observations, we also performed RAPD analysis on six randomly selected yeast strains from other women in this study who had not become symptomatic to determine whether any of these unrelated strains matched strains from those women who became symptomatic.

*Conclusion:* Symptomatic yeast vaginitis is usually due to strains of *C. albicans* already carried in the lower genital tract, underscoring the need to understand regulation of growth and virulence of the organism *in vivo*.


*Infect Dis Obstet Gynecol 2001;9:65–73*

### Candidiasis during pregnancy may result from isogenic commensal strains

**Wayne Daniels[1], Douglas D. Glover[2], Michael Essmann[1] and Bryan Larsen[1]**

[1]*Infectious Disease Research Laboratory, Des Moines University Osteopathic Medical Center, Des Moines, IA*
[2]*Consortium on Reproductive and Developmental Health, Robert C. Byrd Health Sciences Center of the West Virginia University, Morgantown, WV*

***Objective:*** Our laboratory previously demonstrated that asymptomatic vaginal colonization during pregnancy is a factor predisposing patients to subsequent symptomatic vulvovaginal candidiasis. It is unknown whether symptoms result from strain replacement or a change in host relationship to the original colonizing strain. This study was undertaken to determine whether *Candida albicans* isolates from asymptomatic women could be responsible for subsequent symptomatic vaginitis.
***Methods:*** We retained isolates of *C. albicans* from women followed longitudinally through pregnancy, and identified six pairs of cultures from women who were colonized without symptoms and who later became symptomatic (average time 14 weeks). We used a random amplification of polymorphic DNA (RAPD) analysis to determine whether isolates from our study patients were genetically similar or dissimilar.
***Results:*** Analysis of these pairs of yeast strains by RAPD revealed that five of the six women had symptoms apparently due to the same yeast strain that was found initially as a commensal strain. To increase the power of these observations, we also performed RAPD analysis on six randomly selected yeast strains from other women in this study who had not become symptomatic to determine whether any of these unrelated strains matched strains from those women who became symptomatic.
***Conclusion:*** Symptomatic yeast vaginitis is usually due to strains of *C. albicans* already carried in the lower genital tract, underscoring the need to understand regulation of growth and virulence of the organism *in vivo*.

Key words: VAGINITIS, MOLECULAR EPIDEMIOLOGY, PREGNANCY

*Candida albicans* is recognized both as normal flora in the female genital tract and as the causative agent of vulvovaginal candidiasis. What is not clear is whether the vaginal commensal organism increases its virulence to cause candidiasis, the host becomes more permissive toward proliferation of the organism resulting in symptoms, or whether another, possibly more virulent, strain supplants the normal flora organism. The availability of methods of DNA analysis that distinguish between different strains makes it possible to obtain insight into this question.

Soll has recently reviewed the myriad genotyping techniques available to raise epidemiologic investigations of *C. albicans* from the phenotypic to the genotypic level[1]. These techniques have allowed the tracking of transmission of organisms within families, from mothers to infants and within

Correspondence to: Bryan Larsen, PhD, Des Moines University Osteopathic Medical Center, 3200 Grand Avenue, Des Moines, IA 50312. E-mail: bryan.larsen@dmu.edu hospital environments, and the association of genetic fingerprints with virulence and drug resistance. Genotyping approaches are of particular interest to organisms such as *C. albicans*, which can and often do colonize individuals in an asymptomatic fashion. One of the key questions arising from this observation is whether symptomatic candidiasis results from strain replacement. While many epidemiologic studies have been done, including comparison of strains carried simultaneously at different body sites or sequential analysis of strains involved in recurrent infections, the issue of whether symptomatic candidiasis can develop from a strain associated with initially asymptomatic colonization during pregnancy has not been directly addressed.

In the course of a culture-based longitudinal study of vaginal yeast carriage and symptomatic infection during pregnancy, we were able to find pairs of cultures representing asymptomatic colonization, followed at a later time by development of symptoms of vulvovaginal candidiasis[2]. A limited number of pairs of yeast specimens were available, but the discriminating power of random amplification of polymorphic DNA (RAPD) analysis provided sufficient data to determine the relatedness of *C. albicans* cultures present before and during symptomatic vaginitis.

### MATERIALS AND METHODS

More than 300 women who sought obstetric care at the Robert C. Byrd Health Sciences Center and its outreach clinics were the source of vaginal specimens, which were plated onto BIGGY (bismuth sulfite–glucose–glycine–yeast) agar in an effort to identify colonization by *C. albicans*. Each woman was told of our interest in obtaining epidemiologic information on yeast vaginitis in pregnancy and signed a consent form approved by the West Virginia University Institutional Review Board. The demographic composition of this cohort has been described in an earlier paper[2]. All six patients in this study were pregnant at the time of their first culture (range 8–35 weeks' gestation; average gestational age at initial booking was 16 weeks). Two patients were primigravidas and the remaining four patients ranged from gravida 2 to gravida 4; two patients had aborted previously. Three patients had experienced a prior yeast infection and a fourth had experienced recurrent infections. None was symptomatic for yeast or other type of vaginitis (abnormal discharge, itching, burning or malodor) at the time of their initial prenatal examination and all had wet mount and KOH examination to evaluate signs of bacterial vaginosis, trichomoniasis, and candidiasis. All were negative at the initial examination. Each patient was asked to call the clinic in the event of vaginal symptoms. In addition, at each subsequent clinic visit throughout the pregnancy, participants were questioned about symptoms consistent with vulvovaginal candidiasis. Vaginal cultures were obtained from women who developed symptoms so that matched pairs of *C. albicans* isolates could be assembled for this study. Symptomatic individuals were those who complained of or had symptoms of pruritus, inflammation, white curd-like discharge, positive microscopic evidence of vaginal yeast, and positive culture for *C. albicans*.

Cultures obtained from study participants were held at room temperature for at least 72 hours before being discarded as negative. Cultures that developed characteristic brown colonies were held at 4°C until needed. All isolates, in addition to producing brown colonies on BIGGY agar, were shown to have characteristic microscopic morphology and produced germ tubes in human serum, which presumptively classified them as *C. albicans*[3]. A total of seven pairs of cultures representing asymptomatic colonization followed by symptomatic vulvovaginal candidiasis were available. One of these pairs could not be used because the 'asymptomatic' culture became nonviable in storage, leaving six for analysis. These samples were identified by a patient study number (assigned at West Virginia University) and added to the culture collection maintained at Des Moines University.

The six women whose yeast isolates were ultimately included in this study were all white, aged 17–26 years and ranged from 8 to 35 weeks' gestation at their first clinic visit. Two patients were primigravidas and none were private-pay patients, nor did they have alternate sources for medical care. Because of this, all were followed consistently by the study physician.

Just before polymerase chain reaction (PCR) analysis, the paired cultures were again streaked for isolation on BIGGY agar plates, and an individual colony was picked and maximally propagated on a Sabouraud's dextrose agar plate. The growth from this plate was recovered with a sterile inoculating loop and suspended in 293 μl of 50 mmol/l EDTA. Lyticase® (Sigma Chemical Co., St. Louis, MO), 7.5 μl of 20 mg/ml solution, was added and the cell suspension mixed by gentle pipetting. The samples were incubated at 37°C for 1 hour to digest the cell walls. The spheroplasts were brought to room temperature and centrifuged at 13 000 *g*, and the supernatant fluid was discarded.

DNA isolation employed a commercial kit (Wizard Kit®, Promega, Madison, WI), and the manufacturer's directions were followed. The DNA was rehydrated in Tris-EDTA buffer and the absorbance at 260 and 280 nm determined to allow dilution to a final concentration of 10 ng/ml, which served as template for subsequent RAPD analysis.

Our RAPD protocol employed two of the primers from the Ready-To-Go-RAPD® system (Amersham Pharmacia Biotech, Piscataway, NJ): Primer 1 (5′-d[GGTGCG GGAA]-3′) and primer 4 (5′-d[AAGAGCC CGT]-3′). For amplification, 15 μl of sterile distilled water, 5 μl primer, 5 μl of template DNA (50 μg) and one RAPD bead were placed in a reaction tube and reacted in an MJ Minicycler (MJ Research Inc., Watertown, MA). Because the instrument has a heated lid, no mineral oil was added to the reaction mix. The cycling profile was: one cycle at 95°C for 5 min, 40-45 cycles at 95°C for 5 min, 36°C for 1 min, 72°C for 2 min.

The products were applied to a 2% agarose gel containing 1 μg/ml ethidium bromide. A mini-submarine gel format was found to be adequate for separating bands generated from *C. albicans*. A DNA ladder (BioRad, Hercules, CA) with a range of 50–2000 bp was applied to the first lane. The second and third lanes contained the products from primer 1 (the asymptomatic strain in the second lane and the symptomatic strain in the third lane), and the fourth and fifth lanes contained the products from primer 4 (asymptomatic followed by symptomatic).

After electrophoresis, the resulting band patterns were visualized and recorded using the FluorS-Multi-Imager® (BioRad). Analytic densitometry was performed using the Multi-Analyst® software (BioRad). Digitized densitometry scans were exported to a Microsoft Excel® spreadsheet for further analysis.

The densitometry scans of two electrophoretic patterns from the same gel were compared by cross-correlation analysis using a custom program written with LabView® programming software (National Instruments, Austin, TX). The nearer cross-correlation coefficients were to 1.0, the more similar were the two banding patterns. Cross correlation analysis proceeded as follows.

Initially all comparisons were made on adjacent lanes on the same electrophoresis gel, but to compare unrelated strains it was necessary to compare RAPD patterns between gels. Because the number of data points differed between gel images, two data manipulations were performed. First, both electrophoretic patterns were normalized with respect to landmarks on the $x$ axis: that is, the $x$ co-ordinate for each data point was reported as a percentage of the total length between the two tracking dyes. Second, one of the electrophoretic patterns was interpolated so that the data points were matched with respect to the normalized $x$ co-ordinate of the other normalized electrophoretic pattern. Electrophoretic patterns were analyzed using baseline shifts of ±100 lags (lags are data points added by the computer to adjust the length of one $x$ axis).

### RESULTS

Six pairs of cultures representing the vaginal organisms present before symptoms developed and while the patient was symptomatic were analyzed by RAPD analysis. A seventh pair of organisms was identified, but in this case the earliest isolate did not survive in storage. The average length of time between the first *C. albicans* isolation and development of symptoms was 14 weeks (range 9–20 weeks).

Figure 1 shows a composite image of all the PCR products elicited from RAPD analysis. Even without the aid of densitometric analysis it is clear that the banding pattern from the two isolates was identical for both primer 1 and primer 4 in all but one of the six pairs of cultures. The interval

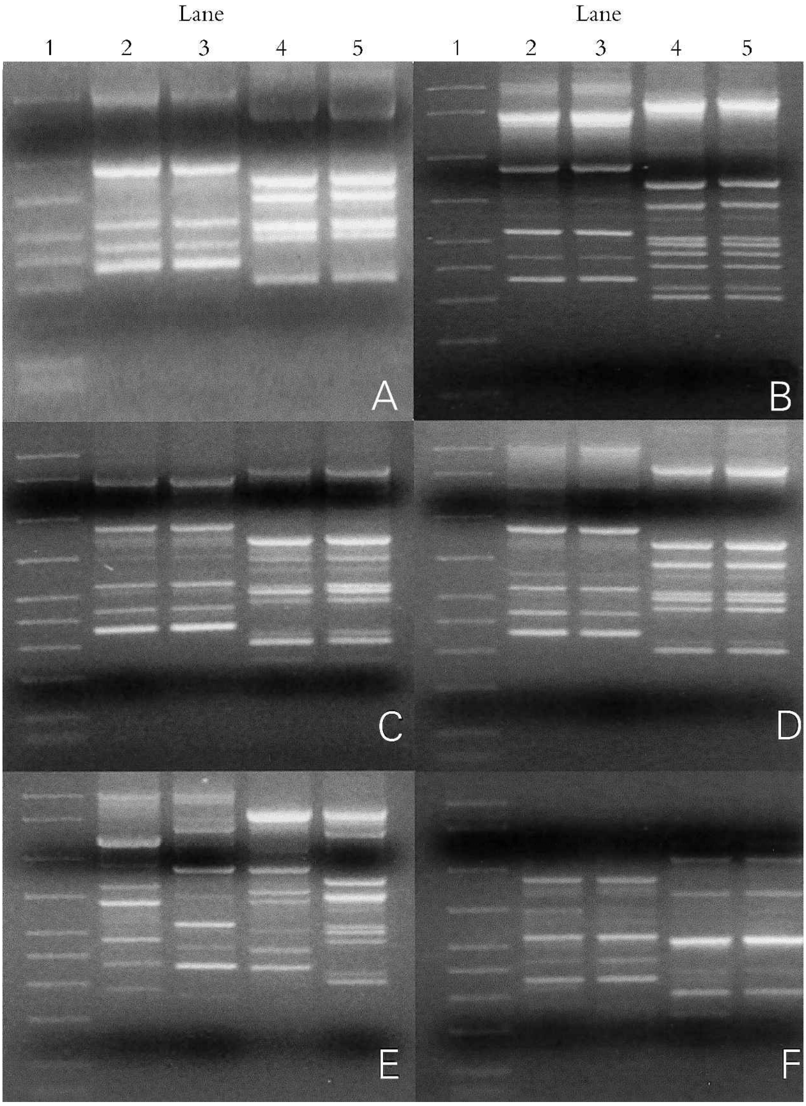

**Figure 1** Electrophoretic pattern of random amplification of polymorphic DNA polymerase chain reaction (RAPD-PCR) products from 12 strains of *Candida albicans* from six patients. Each panel represents the isolate recovered before symptoms and the organism isolated while the patient was symptomatic. The data are from patients GT054 (A), GT151 (B), GT397 (C), GT150 (D), GT377 (E), and GT387 (F). The lanes in each panel are as follows: lane 1: standards (2000 bp and less); lane 2: primer 1 (see Materials and Methods) with target DNA from the asymptomatic patient isolate; lane 3: primer 1 with target DNA from the same patient while symptomatic; lane 4: primer 4 with target DNA from asymptomatic isolate from the same patient; and lane 5: primer 4 with symptomatic isolate between cultures for the non-matching pair was 18 weeks.

Analysis of the PCR products from the 12 yeast isolates in this study generated data on 125 bands with each isolate generating between 11 and 16 bands from the two primers used for the comparison. In order to evaluate this quantity of data, a mathematical method for comparing the banding patterns from the pairs of organisms was employed. Video densitometry provided a more objective method for capturing the RAPD data in a useful form. Figure 2 shows the alignment of two densitometric scans plotted in the LabView software package. This figure consists of the densitometric scan from the two organisms being compared. In this example the two scans appear visually similar when overlaid. The lower plot shows the result of cross-correlation in which the degree of correlation (values ranging from 0–1 with 1 representing perfect correlation) on the

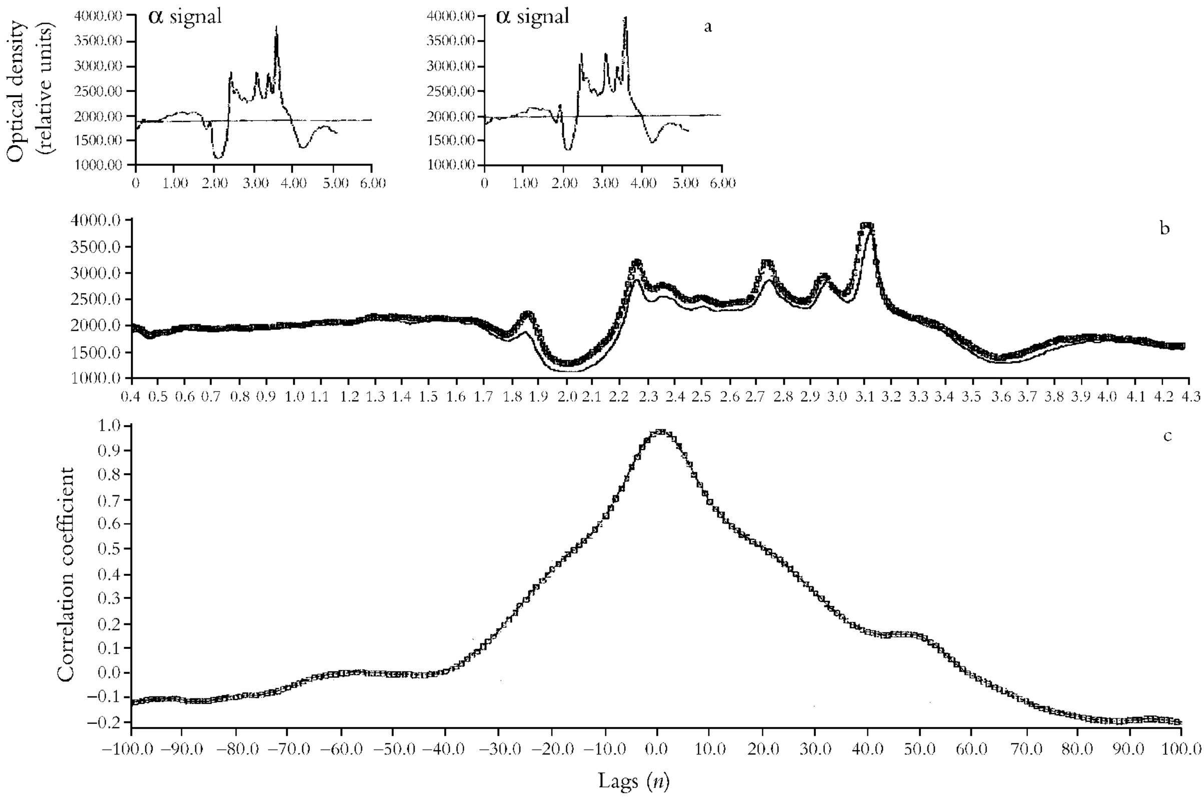

**Figure 2** Graphic results of cross-correlation analysis for isolate GT151. (a) Original densitometric data (*x* axis is distance (relative units); *y* axis is relative optical density units); (b) superimposed traces of asymptomatic and symptomatic isolates; (c) change in cross-correlation coefficient with varying numbers of lags, from −100 to +100 lags (*x* axis is the number of lags; *y* axis is the correlation coefficient)

vertical axis and the number of lags (extra data points used to 'stretch' one of the traces to maximize the alignment). In the example, there was a high degree of correlation. The 'tightness' of the correlation diminished when more than a few lags were inserted.

When cross-correlation was used to determine the relatedness of the RAPD products generated from the remaining *C. albicans* isolates obtained before and after development of symptoms it confirmed that in only one of six cases was there an apparent change in yeast strain from asymptomatic to symptomatic colonization (Table 1).

To assess the meaningfulness of this level of agreement, we selected an additional six yeast isolates randomly from the women in our study who had been found to be colonized without symptoms. Table 2 uses the six isolates from asymptomatic women who later became symptomatic as index cases. These are paired with randomly chosen isolates of *C. albicans* obtained during the course of the longitudinal study previously published with comparisons of the level of discordance by means of cross-correlation analysis[2]. None of the random strains matched the six index isolates, whereas five of six pairs from the initial pairings appeared to be the same (Yates $\chi^2$ $p = 0.019$). This analysis confirms that five of the six women who developed yeast vaginitis symptoms did so as a result of the same organism that colonized them asymptomatically earlier in their pregnancies.

### DISCUSSION

In the past decade, a variety of molecular techniques have emerged which allow a high degree of reliability in epidemiologic tracking of various strains of pathogenic yeasts. These techniques include pulsed field gel electrophoresis[4–6], restriction fragment length polymorphism (RFLP)[6–9], Southern hybridization of specific probes[10,11], RAPD[11–19], and combinations of various methods.

**Table 1** Correlation of electrophoretic patterns of random amplification of polymorphic DNA (RAPD) products from *Candida albicans* isolates before and after development of symptoms

| *Patient number* | *Primer 1 cross-correlation coefficient ($r^2$)* | *Primer 4 cross-correlation coefficient ($r^2$)* |
|---|---|---|
| GT054 | 0.98 | 1.00 |
| GT150 | 0.95 | 0.88 |
| GT151 | 0.98 | 0.97 |
| GT377 | 0.29 | 0.48 |
| GT387 | 0.96 | 0.98 |
| GT397 | 0.96 | 0.97 |

**Table 2** Correlation of presumably unrelated *Candida albicans* strains from asymptomatic women

| *Patient number (index case/ unrelated case)* | *Primer 1 cross-correlation coefficient ($r^2$)* | *Primer 4 cross-correlation coefficient ($r^2$)* |
|---|---|---|
| GT054/GT196 | 0.25 | 0.42 |
| GT150/GT275 | 0.12 | 0.07 |
| GT151/GT158 | 0.24 | 0.29 |
| GT377/GT157 | 0.10 | 0.37 |
| GT387/GT188 | 0.18 | 0.28 |
| GT397/GT199 | 0.13 | 0.11 |

Recently, Soll published an extensive review of the methods and pitfalls associated with DNA fingerprinting of fungal organisms[1]. Strain maintenance over long periods of time appears to be common, but studies that have used very sensitive genotyping methods have revealed the possibility of microevolution among colonizing strains[20]. In addition, the work of Lockhart and colleagues, who investigated recurrent vaginitis, indicated that these women had a repertoire of similar strains that became intermittently predominant strains through a process they termed 'substrain shuffling'[21].

Several reports on *C. albicans* typing have compared different methods[4,22,23], and found RAPD to be a useful method of epidemiologic analysis. Following these organisms within populations is becoming increasingly important because of the prevalence of fungal infections among compromised hosts and other individuals. In addition to understanding the pathogenesis of yeast vaginitis during pregnancy, there is concern about infants, especially premature infants, acquiring *C. albicans* infections. Molecular typing techniques have indicated that one-third of premature infants were colonized and that mother-to-infant transmission of the identical strain occurred in 14% of infants[24]. In another study, there were six cases among 103 newly parturient women in which both mother and infant were colonized by identical strains of *C. albicans*[5].

*C. albicans* and other related yeast are frequently commensal flora on mucosal sites, and it is possible for individuals to carry more than one strain of yeast at a time[25]. It is important to understand if symptoms arise after acquisition of a virulent strain of yeast or if the endogenous organisms can increase their virulence toward the host. Magee's group used an RFLP method to determine that different strains are carried in the vagina and rectum and that recurrent vaginitis is usually due to relapse with the same vaginal strain rather than reinfection from the rectum[9]. Despite this information, it is not clear whether yeast strains present in asymptomatic women are the same ones that are involved in symptomatic infection. Certainly, there is ample evidence that individuals sometimes change strains of yeast and other organisms. Concordance of strains among family members is common[26], as is person-to-person transmission of organisms as documented by Muller and colleagues in an HIV-infected family[27], implying that the development of symptomatic infection could be the result of acquiring a new yeast strain.

The present study was possible because we were engaged in following women longitudinally during pregnancy with a culture for *C. albicans* when they booked for obstetric care and then again if they developed symptoms consistent with yeast vaginitis. Clearly, a larger number of pairs of strains would have been desirable for evaluation, but because of the prospective nature of the culture collection and our inability to predict which women would become symptomatic, we believe that we would need to follow an additional 50 women through pregnancy to elicit just one more pair of cultures. Based on RAPD analysis, the majority of vulvovaginitis cases were apparently due to the same strain of organism that was present many weeks before the symptoms arose. These findings suggest that the changing relationship between the host and microorganism during the progression of the pregnancy is probably more important in the development of symptoms than is strain replacement or even a genetic change in the commensal organism. We believe this study, and potentially others like it, can benefit from the mathematical method of expressing the comparison of two gel patterns as a single correlation coefficient. Additional work will be required to ascertain the level of sensitivity to generic differences represented by the cross-correlation technique, but clearly it distinguishes paired samples from random samples.

One limitation of our approach was that only one colony was isolated from each of the *C. albicans*-positive cultures, which was done to ensure purity of the isolates we tested. Although in the 12 positive cultures forming the core of this study the morphotypes of the colonies on primary plating media appeared identical, a small possibility exists that we could have missed isolating a second strain that might have been present in the same culture. Because the occurrence of two concomitant strains of yeast in the same vaginal culture is relatively uncommon, and because yeast causing symptoms are generally thought to be in numerical abundance, the likelihood of having missed a second yeast strain in this cohort of patients is considered very low. On the other hand, the one discordant pair of yeast we discovered could have been the result of having two strains simultaneously in the same patient and our having happened to isolate the nonidentical strain in the 'symptomatic' culture.

Until obtaining DNA sequence data becomes the standard method for genotyping there is a possibility that less sensitive analytical techniques such as RAPD will miss mutations and microevolution occurring in organisms carried for a long period of time. A study by Metzgar and co-workers[16] found small changes in nucleotide sequence data during the course of fluconazole treatment in some HIV-infected patients, raising the possibility of genetic changes in the short time from isolation of a commensal organism to the time that symptoms develop. The study of Metzgar and co-workers involved a much more in-depth analysis of the genotypic characteristics of their organisms than our RAPD analysis. Thus, small mutational events could have been more readily apparent in their work. In fact, their work suggests that during therapy the patients in their study probably retained the same organisms, but that these organisms were undergoing microevolution.

Despite the fact that there are more sensitive methods of genetic characterization of microorganisms, the evidence gained from the use of RAPD analysis provides a good basis for believing that in most cases vaginal symptoms may result from the same organism present prior to the development of symptoms. This raises a further question of whether a change in host physiology or in regulation of virulence attributes of the microorganism (or a combination of both) is responsible for genesis of symptoms in these patients. While the occurrence of mucocutaneous candidiasis in HIV patients emphasizes the importance of changing immune status in fungal pathogenesis, there is also a growing recognition that microorganisms have the ability to alter their aggressiveness toward the host in response to microenvironmental cues. Environmental factors such as glucose concentration[28], iron[29], or hormonal factors[30] may play a role in modulating *C. albicans* virulence. This fact, coupled with the recognition that the same organism which colonizes the vaginal epithelium may subsequently cause vaginal irritation, underscores the importance of understanding the regulation of microbial physiology and the role of host factors in microbial regulation.

### ACKNOWLEDGEMENT

The assistance of Joseph Weir, PhD, in analyzing densitometry patterns aided materially in the presentation of this work.

### REFERENCES

1. Soll DR. The ins and outs of DNA fingerprinting the infectious fungi. *Clin Microbiol Rev* 2000; 13:332–70
2. Glover DD, Larsen B. Longitudinal investigation of *Candida* vaginitis in pregnancy: role of superimposed antibiotic use. *Obstet Gynecol* 1998;91: 115–18
3. Silva-Hunter M. Yeasts of medicinal importance. In Lenette EH, ed. *Manual of Clinical Microbiology,* 3rd edn. Washington, DC: American Society for Microbiology Press, 1980:562–76
4. Bostock A, Khattak MN, Matthews R, Burnie J. Comparison of PCR fingerprinting, by random amplification of polymorphic DNA, with other molecular typing methods for *Candida albicans*. *J Gen Microbiol* 1993;139:2179–84
5. Willinger B, Berger A, Li L, *et al.* Epidemiological analysis of *Candida* yeast by pulsed-field gel electrophoresis. *Mycoses* 1994;37:401
6. Dib JC, Dube M, Kelly C, *et al.* Evaluation of pulsed-field gel electrophoresis as a typing system for *Candida rugosa*: comparison of karyotype and restriction fragment length polymorphisms. *J Clin Microbiol* 1996;34:1494–6
7. McCullough MJ, Clemons KV, Stevens DA. Molecular epidemiology of the global and temporal diversity of *Candida albicans*. *Clin Infect Dis* 1999;29:1220–5
8. Ruiz-Diez B, Martinez V, Alvarez M, *et al.* Molecular tracking of *Candida albicans* in a neonatal intensive care unit. Long-term colonizations versus catheter-related infections. *J Clin Microbiol* 1997; 35:3032–6
9. Stein GE, Sheridan VL, Magee BB, Magee PT. Use of rDNA restriction fragment length polymorphisms to differentiate strains of *Candida albicans* in women with vulvovaginal candidiasis. *Diagn Microbiol Infect Dis* 1991;14:459–64
10. Joly D, Pujol C, Schroppel K, Soll DR. Development of two species-specific fingerprinting probes for broad computer-assisted epidemiological studies of *Candida tropicalis*. *J Clin Microbiol* 1996; 34:3063–71
11. Powderly WG, Robinson K, Keath EJ. Molecular epidemiology of recurrent oral candidiasis in human immunodeficiency virus-positive patients; evidence for two patterns of recurrence. *J Infect Dis* 1993;168:463–6
12. Gyanchandani A, Khan ZK, Farooqui N, *et al.* RAPD analysis of *Candida albicans* strains recovered from different immunocompromised patients (ICP) reveals an apparently non-random infectivity of the strains. *Biochem Mol Biol Int* 1998;44:19–27
13. Holmberg K, Feroze F. Evaluation of an optimized system for random amplified polymorphic DNA (RAPD)-analysis for genotypic mapping of *Candida albicans* strains. *J Clin Lab Anal* 1996; 10:59–69
14. Howell SA, Anthony RM, Power E. Application of RAPD and restriction enzyme analysis to the study of oral carriage of *Candida albicans*. *Lett Appl Microbiol* 1996;22:125–8
15. Lehmann PF, Lin D, Lasker BA. Genotypic identification and characterization of species and strains within the genus *Candida* by using random amplified polymorphic DNA. *J Clin Microbiol* 1992;30: 3249–54
16. Metzgar D, van Belkum A, Field D, *et al.* Random amplification of polymorphic DNA and microsatellite genotyping of pre- and post-treatment isolates of *Candida* spp. from human immunodeficiency virus-infected patients on different fluconazole regimens. *J Clin Microbiol* 1998;36: 2308–13
17. Reiss E, Tanaka K, Bruker G, *et al.* Molecular diagnosis and epidemiology of fungal infections. *Med Mycol* 1998;36(Suppl 1):249–57
18. Steffan P, Vazquez JA, Boikov D, *et al.* Identification of *Candida* species by randomly amplified polymorphic DNA fingerprinting of colony lysates. *J Clin Microbiol* 1997;35:2031–9
19. Zeng S, Wu LC, Lehmann PF. Random amplified polymorphic DNA analysis of culture collection strains of *Candida* species. *J Med Vet Mycol* 1996;34:293–7
20. Lockhart S, Fritch JJ, Meier SA, *et al.* Colonizing populations of *Candida albicans* are clonal in origin but undergo microevolution through C1 fragment reorganization as demonstrated by DNA fingerprinting and C1 sequencing. *J Clin Microbiol* 1995; 33:1501–9
21. Lockhart SR, Reed DB, Pierson CL, Soll DR. Most frequent scenario for recurrent *Candida* vaginitis is strain maintenance with 'substrain shuffling': demonstration by sequential DNA fingerprinting with probes Ca3, Ca1 and CARE2. *J Clin Microbiol* 1996;34:767–77
22. Clemons KV, Feroze F, Holmberg K, Stevens DA. Comparative analysis of genetic variability among *Candida albicans* isolates from different geographic locales by three genotypic methods. *J Clin Microbiol* 1997;35:1332–6

23. Dahl KM, Keath EJ, Fraser VJ, Powderly WG. Molecular epidemiology of mucosal candidiasis in HIV-positive women. *AIDS Res Hum Retrovir* 1997;13:485–91
24. Waggoner-Fountain LA, Walker MW, Hollis RJ, *et al.* Vertical and horizontal transmission of unique *Candida* species to premature newborns. *Clin Infect Dis* 1996;22:803–8
25. Xu J, Boyd CM, Livingston E, *et al.* Species and genotypic diversities and similarities of pathogenic yeasts colonizing women. *J Clin Microbiol* 1999;37:3835–43
26. Mehta SK, Stevens DA, Mishra SK, *et al.* Distribution of *Candida albicans* genotypes among family members. *Diagn Microbiol Infect Dis* 1999;34:19–25
27. Muller FM, Kasai M, Francesconi A, *et al.* Transmission of an azole-resistant isogenic strain of *Candida albicans* among human immunodeficiency virus-infected family members with oropharyngeal candidiasis. *J Clin Microbiol* 1999;37:3405–8
28. Hostetter MK, Lorenz JS, Preus L, Kendrick KE. The iC3b receptor on *Candida albicans*: subcellular localization and modulation of receptor expression by glucose. *J Infect Dis* 1990;161:761–8
29. Ramanan N, Wang Y. High affinity iron permease essential for *Candida albicans* virulence. *Science* 2000;288:1062–4
30. Zhang XQ, Essmann M, Burt E, Larsen B. Estrogen effects on *Candida albicans*: A potential virulence-regulating mechanism. *J Infect Dis* 2000;181:1441–6

RECEIVED 08/11/00; ACCEPTED 01/18/01
